# Notes and Observations in General Practice

**Published:** 1868-09

**Authors:** John Forman

**Affiliations:** Chicago


ARTICLE XXXV.

### NOTES AND OBSERVATIONS IN GENERAL PRACTICE.

By JOHN FORMAN, M.D., of Chicago.

### THE CARBO-SULPHITES IN PUERPERAL SEPTICAMÆ.

The following case seems an excellent example of the value of the carbo-sulphites in the treatment of puerperal septicamæ, in the twofold sense of a deoxidizing and catalytic agent.

On the 22d July, I was requested by Dr. Rawson to join him where he was in attendance upon Mrs. C., who was in labor with her sixth child. Dr. R. stated that he had been in attendance four hours, and although labor-pains had continued regular, very frequent, and vigorous during the whole of that time, not the slightest progress had been made. At 1 P.M. I found a polypus protruding from the vagina, and traced its connection to the posterior lip of the *os uteri.* In shape and size it resembled a Jargonelle pear. The *os uteri* was fully dilated and dilatable; membranes intact; head presenting, face to sacrum; occiput tightly wedged, and resting on the *os pubis;* repeated efforts with the hand to relieve it were futile. After waiting three-quarters of an hour, and observing the patient's strength rapidly diminishing, from the pressure of vigorous pains every two minutes, without any progress, applied Prof. Simpson's long forceps, and, after exercising considerable force, succeeded in delivering a large female child. Alarming hemorrhage immediately followed; the placenta was at once removed, the uterus grasped with the hand internally, and ice applied over the pubic region; free bleeding still continued, which was traced to the polypus, which had been lacerated. This was secured and ice-water cloths applied, renewing them at short intervals. In the course of an hour and a-half bleeding entirely ceased, leaving our patient much exhausted, frequently bordering on syncope. Stimulants and ice-water were freely administered. At 6 P.M., she began to rally; pulse small and quick; skin hot and perspiring; intense thirst; temperature of room 96.° To continue stimulants. Milk and ice.

*23d, 8 A.M.*—Patient feels very uncomfortable; slept none; has headache, thirst, and skin hot; temperature in axilla 110°; temperature of room 92°; abdomen distended and tympanitic; uterus not contracted, very tender on pressure, particularly over right iliæ; no after-pains; no lochial discharge; fœtor very strong. The uterus was injected with a quart of tepid water, containing a drachm of carbolic acid crystals, and repeated three times a day. A scruple of sulphite of soda, six grains of crystallized carbolic acid, with half a drachm of liquor secali, were ordered every four hours, with stimulants and a nourishing regimen.

Little improvement took place until the evening of the 25th, when uterine discharge appeared, and the pulse and temperature of the skin simultaneously fell. The carbo-sulphite mixture was continued, with a good, nourishing regimen; the deodorizing injection used twice a day, for two days more; when our patient was convalescent.

*Remarks.*—Although puerperal septicamæ never reached the typhoid stage in this case, the strong probability is, that if the case had been left to nature, or treatment of a less decided character employed, such an unhappy result would have occurred, from the fact of two fruitful sources being present: 1st. A patient exhausted in the extreme after a vigorous, tedious labor, followed with alarming hemorrhage. 2d. The entire absence of *post partem* uterine contraction (no doubt resulting from the double cause of exhaustion, and cold applications used to stem fast-ebbing life), with the sequelæ of decomposing uterine coagula, this in course becoming absorbed and quickly firing the train of deadly puerperal typhoid.

The carbo-sulphites bid fair to become the physician's sheet-anchor in zymosis, as Prof. Lister's dressings are to conservative surgery.

